# Dysregulation of Astrocyte–Neuronal Communication in Alzheimer’s Disease

**DOI:** 10.3390/ijms22157887

**Published:** 2021-07-23

**Authors:** Carmen Nanclares, Andres Mateo Baraibar, Alfonso Araque, Paulo Kofuji

**Affiliations:** Department of Neuroscience, University of Minnesota, Minneapolis, MN 55455, USA; cperezde@umn.edu (C.N.); barai007@umn.edu (A.M.B.); araque@umn.edu (A.A.)

**Keywords:** Alzheimer’s disease, gliotransmission, calcium, amyloid plaques, glia

## Abstract

Recent studies implicate astrocytes in Alzheimer’s disease (AD); however, their role in pathogenesis is poorly understood. Astrocytes have well-established functions in supportive functions such as extracellular ionic homeostasis, structural support, and neurovascular coupling. However, emerging research on astrocytic function in the healthy brain also indicates their role in regulating synaptic plasticity and neuronal excitability via the release of neuroactive substances named gliotransmitters. Here, we review how this “active” role of astrocytes at synapses could contribute to synaptic and neuronal network dysfunction and cognitive impairment in AD.

## 1. Introduction

The main independent variable linked to the risk of suffering Alzheimer’s disease (AD) is aging [1,2,3]. AD is the most common neurodegenerative disorder with the contribution of multiple cell types throughout the central nervous system. Most cases are sporadic, and only 2–5% of patients have a genetic familial background. Alois Alzheimer in 1907 first described the histopathological characteristics of the disease with the presence of senile plaques (formed by the extracellular aggregation of amyloid β (Aβ) protein) and neurofibrillary tangles (formed by the destructuring of the microtubules due to hyperphosphorylation of the tau protein) [4,5,6].

Even though the disease was described more than a century ago, its underlying causes are still unknown today. It is thought to be a disease of multifactorial etiology, with numerous risk factors, among them genetic, age, sex, educational level, and diet. The systematic biochemical investigation of the brains of patients with AD in the late 1960s and early 1970s led to the cholinergic hypothesis [7]. Thus, it was proposed that the degeneration of cholinergic neurons in the basal forebrain and the associated loss of cholinergic neurotransmission in the cerebral cortex and other areas contribute significantly to the deterioration in cognitive function seen in patients with AD [7,8]. Later, the observation of accumulation and deposition of oligomeric or fibrillary Aβ peptides in AD patient brains popularized the amyloidogenic hypothesis as the primary cause of AD and remains a mainstream concept [4,9,10]. The central role of the Aβ peptide in AD pathogenesis has been supported by the fact that familial forms of the disease are caused by its overproduction. The Aβ peptide is produced by processing of the amyloid-β protein precursor (APP) through sequential cleavage by β- and γ-secretases in the amyloidogenic pathway [10]. Indeed, the majority of the AD animal models used today are mice transgenic for human APP and Aβ [11]. Likewise, the contribution of microtubule-associated protein tau in AD pathology has also been proposed [12,13]. In this hypothesis, the presence of abnormally high levels of tau protein in the hyperphosphorylated state promotes the production of toxic oligomeric tau and paired helical filaments, which further assemble into toxic neurofibrillary tangles. Thus, it is clear that more studies are needed to explain the etiopathogenesis of AD, which is a critical step for developing rational treatments.

The most predominant and striking sign in an AD patient is the progressive decline in cognition, which is primarily due to the loss of neurons and synapses in the hippocampal formation and related areas [14]. As expected, research on AD has focused mainly on neuronal cells, which allowed great insights on the neurocentric molecular pathways involved in these processes, such as protein degradation systems, post-translational modifications, and interactions with cytoskeletons. However, in the last three decades, growing evidence suggests that glial cells, in particular astrocytes, have a contributing role in AD pathophysiology. Astrocytes in the human brain play key roles in numerous functions within the central nervous system such as structural support, ionic balance of the extracellular space, neurotransmitter clearance at synapses, and modulation of synaptic signaling. Thus, it is hardly surprising that astrocytes have been implicated in the pathology of several neurodegenerative diseases, including AD. Numerous excellent reviews have discussed the role of astrocytes in AD in the context of altered inflammatory processes [15,16,17], amyloid clearance [18], and neurovascular coupling [19,20]. The present review focuses instead on the role of dysregulated bidirectional communication of neurons and astrocytes in AD pathology.

## 2. Basic Structure and Function of Astrocytes

The term astrocyte was created to describe star-shaped glial cells detected in histological brain specimens [21]. Glia often was considered to vastly outnumber neurons in the brain but a re-examination of various counting methods place glia/neuron ratios closer to 1:1 in the human brain [22]. Traditionally, two major classes of astrocytes have been distinguished in histological sections of the central nervous system (CNS) based on their morphology and distribution [23]. The fibrous astrocytes are mainly in the white matter with few processes, and protoplasmic astrocytes are mostly found in gray matter and characterized by their intricate morphology with complex branching processes. More recently, RNA and proteomic analysis have identified several distinct astrocyte subtypes in diverse brain areas reflecting the distribution of morphologically and physiologically distinct astrocyte populations [24,25,26].

The tridimensional reconstruction of individual protoplasmic astrocytes in the rat hippocampus showed that astrocyte cell bodies are evenly spaced, and their processes overlap only minimally, creating a ‘tiling” of astrocytes [27,28]. This seems to be the case in other brain regions as well [29]. Furthermore, a single astrocyte in the rat hippocampus is estimated to occupy a territory of 66,000 µm^3^ of neuropil and contact over 140,000 synapses [27], placing a single astrocyte in a favorable position to modulate the activity of a vast neuronal network. Astrocytes in the human cortex have even larger territories and complex structural features [30]. Compared to rodents, the volume of human astrocytes is about 27-fold greater with a 2.5-fold increased diameter and each astrocyte is estimated to contact up to ≈2,000,000 synapses [30].

Traditionally, an important function attributed to astrocytes has been the removal or clearance of synaptic neurotransmitters such as glutamate or GABA [31,32]. Other “supporting” functions attributed to astrocytes comprise their participation in neural development, the buffering of extracellular potassium, and the regulation of blood flow [33,34,35,36,37].

Comparatively to neurons, the functional properties of astrocytes have received little attention. One major reason for this is the lack of active voltage-gated conductances in astrocytes. Electrophysiologically, astrocytes under voltage-clamped conditions display a quasi-linear voltage–current relationship with a cellular membrane almost exclusively permeable to potassium ions [38]. This potassium selective membrane is endowed by the expression of large amounts of inwardly rectifying potassium (Kir) channels, conferring astrocytes with their characteristic low input resistance and membrane potential close to the predicted equilibrium potential for transmembrane potassium. Molecular cloning of Kir channels in astrocytes shows that they are mainly weakly inwardly rectifying Kir4.1 channels [39,40]. Another major conductance found in astrocytes is connexin 43, which provides intercellular electrical coupling among astrocytes [41]. These electrophysiological features of astrocytes led to the hypothesis of “potassium spatial buffering”. In this hypothesis, when a localized release of potassium occurs due to enhanced neuronal activity, the excess extracellular potassium would enter the astrocytic syncytium and be expelled in other areas where the extracellular potassium is low [38].

## 3. Astrocytic Release of Gliotransmitters

**Figure 1:**
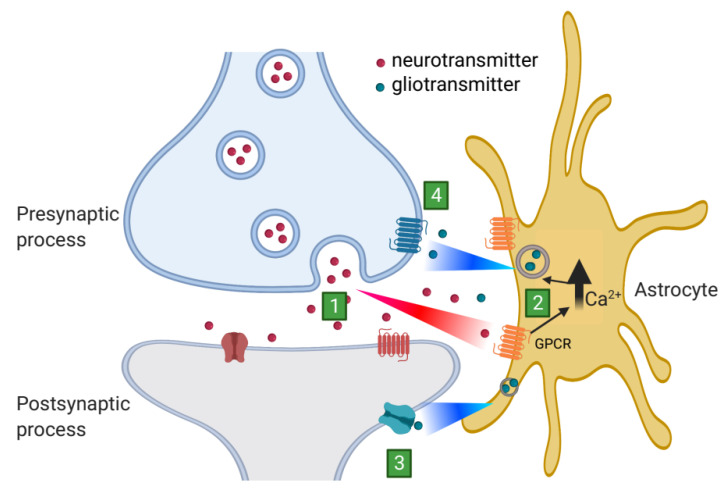
Schematic representation of a tripartite synapse. The tripartite synapse is composed of presynaptic and postsynaptic processes with astrocytic processes enwrapping the synapses. (1) The release of neurotransmitter from the presynaptic terminal acts on the postsynaptic terminal as well as with astrocytic receptors mediating intracellular calcium elevation via G-protein coupled receptors (GPCRs). (2) Then, calcium elevation triggers the release of gliotransmitters that bind with the postsynaptic terminal receptors (3) or presynaptic receptors (4) to modulate synaptic transmission.

Mainly due to the development of organic and genetically encoded calcium indicators and advances in imaging techniques, it has become clear that astrocytes have an “active” role in modulating the activity of neuronal circuits [42,43,44,45]. During synaptic activity, the release of neuronal transmitters leads to changes in intracellular calcium activity in astrocytes. This is so because astrocytes express a myriad of G-protein-coupled receptors (GPCRs) that respond to neurotransmitters through the activation of inositol triphosphate type 2 receptors (IP3R2), which mediate calcium release from the endoplasmic reticulum [46,47]. Such calcium elevations have been observed both in the astrocytic processes or microdomains and in their somata [37,48,49]. An outcome of the intracellular calcium changes in astrocytes is the release of neuroactive molecules or “gliotransmitters” [50]. Among them are ATP, glutamate, D-serine, and GABA [47,50,51,52,53,54,55,56,57,58,59,60]. Through the release of gliotransmitters, astrocytes have been found to modulate neuronal activity and synaptic transmission in several brain areas [47,55,61,62] and to impact animal behavior [63,64,65]. This bidirectional exchange of information between astrocytes and neurons is embodied in the concept of the tripartite synapse that includes astrocytes as integral elements of synaptic function along with presynaptic and postsynaptic processes (Figure 1) [42,44,47].

Remarkably, gliotransmitters modulate many forms of synaptic plasticity at various temporal and spatial scales [47,66]. Astrocytic modulation of presynaptic or postsynaptic neuronal terminals has been associated with short-term synaptic potentiation or depression [66]. Astrocytic modulation of long-lasting synaptic changes as long-term potentiation (LTP) and long-term depression (LTD) [47] is also extensively documented.

## 4. Reactive Astrocytes in Neurodegenerative Diseases

A hallmark sign of neurodegenerative disease is reactive gliosis with astrocytic morphological and functional changes [67,68,69]. Astrocyte reactivity is initially characterized by the hypertrophy of soma and processes triggered by inflammatory molecules [33,68]. In addition, there is an increased expression of intermediary filament proteins such as Glial Fibrillary Acidic Protein (GFAP) or vimentin [70,71]. Many studies have shown that in AD, there is a prominent presence of reactive astrocytes, in particular, surrounding amyloid plaques [72,73]. They are detected at the early phases of AD even before neuronal death and are found ubiquitously throughout disease progression [74]. In addition to morphological changes, they display graded reactivity with heterogeneous gene expression, morphology, and function [74,75]. Concerning this review, an important consequence of astrocyte reactivity in disease is the abnormal gliotransmitter release [76].

## 5. Altered Astrocyte Calcium Excitability in AD

There is great consensus among all available studies that in parallel with morphological changes and enhanced GFAP expression, spontaneous calcium signals in astrocytes are heightened in brains of AD animal models [77,78,79,80,81,82]. This increase appears to be independent of neuronal activity, since the blockade of neuronal activity with the sodium channel blocker tetrodotoxin does not instigate changes in spontaneous calcium activity [77,78,79]. This is in contrast to what is observed in normal aging, where no changes or even fewer spontaneous calcium oscillations occur [83,84].

More specifically, Kuchibhotla et al. found that astrocytes in the adult APP/PS1 mice (6 to 8 months old) with cortical amyloid plaques exhibited a significant increase in calcium transients with events synchronously coordinated across long distances and uncoupled from neuronal activity [78]. Likewise, Takano et al. using mice expressing the Swedish mutation of the APP gene showed that astrocytes exhibit a higher frequency of spontaneous oscillations even before the appearance of amyloid plaques [81]. Mechanistically, the increase in calcium activity in astrocytes may be mediated through the upregulation of neurotransmitter receptors in astrocytes such as the purinergic receptor P2Y1R [80,85], α7 nicotinic acetylcholine receptors (α7 nAChR), or metabotropic glutamate receptors mGluR5 [86,87]. Thus, the application of Aβ25–35 peptide, a neurotoxic Aβ fragment present in AD patients, upregulates calcium transients in primary cortical astrocytes, which can be blocked with P2 receptor antagonists [88]. Likewise, studies in vivo show that the enhanced calcium activity of astrocytes near Aβ plaques in APPPS1 mice is reduced after P2 purinoreceptor blockade [77].

Alternatively, Aβ plaques may bind and activate/inactivate α7 nAChR in astrocytes leading to hyperactivity in calcium signaling. In the human hippocampus and entorhinal cortex, α7, but not α4, subunit immunoreactivity is associated with astrocytes [86]. Moreover, a higher proportion of α7 expressing astrocytes was found in samples from AD patients compared with age-matched controls [86,87]. Evidence of an effect of Aβ plaques or oligomers on α7 nAChRs in astrocytes was obtained in hippocampal slices. Astrocytic calcium elevations can be elicited with nicotine and Aβ1-42 application in hippocampal slices [89]. These responses were present even in the presence of a sodium blocker, suggesting a neuronal-independent effect [89]. This scenario is supported by the fact that the incubation of rat hippocampal slices with Aβ1-42 at a concentration as low as 200 pM induces an increase in astrocytic calcium transients that is dependent on α7 nAChR expression or activity [90].

**Figure 2:**
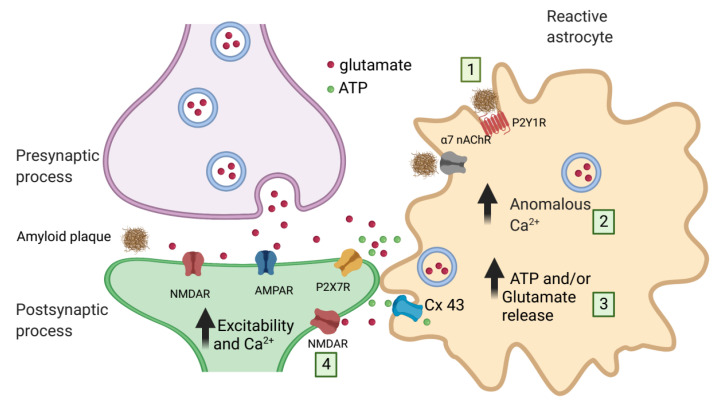
Schematic representation of ATP and glutamate release from astrocytes in AD pathology. (1) Amyloid plaques react with P2Y1R or α7 nAChRs to evoke anomalous and increased intracellular calcium increases in astrocytes (2). These calcium increases induce the release of glutamate and/or ATP via vesicle release or hemichannel Cx43 release (3). ATP and glutamate act on P2X7Rs or NMDARs to increase the excitability and calcium concentrations of neurons (4). Arrows indicate increases.

Given that homomeric α7 nAChR displays high permeability to calcium [91], stimulation of α7 nAChRs in astrocytes could lead to enhanced calcium activity in astrocytes via influx from extracellular sources. However, it has been shown that the increase in intracellular calcium in astrocytes via α7 nAChR signaling involves release from intracellular stores [92]. Since astrocytes can release gliotransmitters in response to intracellular calcium elevations, the upregulation of α7 nAChRs in astrocytes in AD may trigger the dysregulated release of gliotransmitters such as glutamate to ultimately induce neurotoxicity [79] (Figure 2).

Alternatively, the appearance of calcium hyperactivity in astrocytes may also be due to enhanced expression of the metabotropic glutamate receptor mGluR5 in astrocytes. Astrocytes detect glutamatergic transmission through mGluR5, and its activation regulates multiple forms of astrocyte–neuronal communication [93]. The relevance of mGluR5 signaling in astrocytes in adult brains remains somewhat controversial [94]; however, there seems a consensus of its upregulation in reactive astrocytes [95,96,97].

Thus, it is not surprising that the upregulation of mGluR5 is detected in astroglial cultures exposed to β-amyloid or in astrocytes in animal AD models and in postmortem human tissues [98,99,100,101,102]. Astrocytic mGluR5 activation by Aβ plaques leads to sustained calcium oscillations in the reactive astrocytes [103], which may trigger the release of intracellular glutamate, thus enhancing the neuronal excitability leading to excitotoxicity.

Several studies have also implicated the hyperactivity of calcium activity in astrocytes to the dysregulated function of neuronal networks in AD mouse models [104,105,106]. It has been hypothesized that this calcium hyperactivity of astrocytes networks may contribute to cognitive deficits observed in AD. Indeed, diminishing astrocyte calcium signaling pharmacologically or by astrocyte-specific genetic deletion (*Ip3r2*^−/−^) normalizes the neuronal network dysfunction and improves spatial memory in the APP/PS1 mouse model [80].

Notably, Richetin et al. found an accumulation of a tau isoform (3R) in hilar astrocytes of the dentate gyrus of patients afflicted with AD [107]. The overexpression of tau 3R isoform in dentate gyrus astrocytes in mice induced abnormal mitochondrial function in astrocytes and impaired synaptic and network activity in the hippocampus [107]. Behaviorally, tau accumulation in astrocytes also led to impairments in spatial memory tests. These results compellingly indicate that the accumulation of tau in astrocytes contributes to the pathogenesis of AD disease. So far, the impact of tau accumulation in astrocytic calcium excitability communication is unknown.

## 6. Altered Glutamate Release and Uptake from Astrocytes in AD

Glutamate is the major excitatory transmitter in the central nervous system and has a large array of physiological functions including learning and memory. Several families of glutamate receptor proteins have been identified: N-methyl-D-aspartate (NMDA) receptors, α-amino-3- hydroxy-5-methyl-4-isoxazole propionic acid (AMPA) receptors, kainate receptors, and metabotropic receptors [108].

Glutamate concentrations in the extracellular space are kept at low levels and tightly controlled by several mechanisms at the synapse. Perturbations to this regulatory system can lead to deleterious effects such as excess of extracellular glutamate, which can induce hyperexcitability in post-synaptic neurons to the point of excitotoxicity and cell death (cytotoxicity). It is usually accepted that the glutamate transporter activity of astrocytes is the primary mechanism responsible for clearing extracellular glutamate at synapses [109]. The major type of glutamate transporter expressed in astrocytes is the sodium-dependent symporter GLT-1 (GLT-1), since it is responsible for around 90% of astrocytic glutamate uptake in the brain [110], expressing at levels four or six times higher than the glutamate aspartate Transporter 1 (GLAST1) [111]. Moreover, the dysregulation of GLT-1 has been linked to neuronal cell death and neurological disorders, and the GLT-1 knockout mouse has a phenotype of lethal spontaneous seizures and significant neuronal loss [112].

Chronic glutamate excitotoxicity has been hypothesized to play a role in numerous neurodegenerative diseases including amyotrophic lateral sclerosis, AD, and Huntington’s disease [113]. The bulk of evidence indicates alterations in expression or subcellular localization of GLT-1 in AD [114,115,116,117]. Lower expression of GLT-1 mRNA in AD hippocampus and a 30% reduction of GLT-1 immunoreactivity in AD frontal cortex have been observed, with no concomitant decreases in GLAST [118]. Autopsied AD hippocampus showed decreased GLAST and GLT-1 mRNA expression with altered localization of the corresponding proteins into neurofibrillary tangles [119]. In line with the above-mentioned findings, functional studies indicate a reduction in glutamate uptake activity in AD brains. In autopsies of AD patients, with the decrease of the GLT immunoreactivity, there is also a decrease in glutamate transporter activity [118,120]. Reducing expression of GLT-1 in the AβPPswe/PS1ΔE9 mice accelerated cognitive deficits reminiscent of AD [121]. Accordingly, the overexpression of GLT-1 in the APP Swedish mice improved their cognitive functions and decreased pathology [122]. A more direct demonstration of altered glutamate homeostasis in AD mouse models is provided by Hefendehl et al. using in vivo two-photon imaging and the glutamate sensor iGluSnFR [123]. In the APPPS1 transgenic mice, the glutamate sensor shows that around Aβ plaques, there are high spontaneous and anomalous glutamate fluctuations [123]. Moreover, the dynamics of glutamate build-up and clearance upon sensory stimulation are also altered in these mice with a reduction of GLT-1 expression around Aβ plaques [123]. Conversely, AD patients with preserved cognitive function (no dementia) showed enhanced GLT-1 expression in comparison to controls (AD patients with dementia) [124]. Collectively, these studies demonstrate the potential importance of temporal changes of GLT-1 expression and localization in AD.

Astrocytes are not only involved in glutamate uptake via transporter activity, but they can also release glutamate as a gliotransmitter, most likely via a vesicular mechanism [125]. Glutamate released by astrocytes binds to either presynaptic metabotropic glutamate receptors or extrasynaptically-located postsynaptic NMDA receptors (NR2B) [126,127]. The latter evokes the so-called slow inward currents (SICs), which can be discriminated from the synaptic potentials by their much slower temporal kinetics [53,128,129,130,131]. The depolarizing action of these SICs is hypothesized to modulate neural excitability to affect neuronal action potential firing [132]. Moreover, because single astrocytes are near a large number (≈100) of neurons [29], SICs can be generated in many adjacent neurons to promote the synchrony of neuronal firing [53,133,134].

Overall, several studies report an increase in astrocytic-mediated SICs in animal or experimental models of AD [82,135,136]. For example, in rat hippocampal slices, application of the Aβ1-42 peptide not only induces increases in astrocytic calcium activity but also induces the increase of SIC frequency in CA1 pyramidal neurons [79] or cortical cultures [137] (Figure 2). Moreover, in the Tg2576 AD mouse model for Aβ over-production and accumulation, spontaneous astrocytic calcium elevations and SICs were also found [79]. More indirect measurements using fluorometric methods also show the effect of Aβ in eliciting glutamate release from astrocytes in hippocampal slices [135] or cortical cultures [88,136].

The heightened release of glutamate from astrocytes may underlie the changes in synaptic function and plasticity observed in animal models of AD. Activation of extrasynaptic NMDARs triggered by the glutamate release from astrocytes in response to Aβ peptide is followed rapidly by a decrease in miniature excitatory postsynaptic currents (mEPSC), representing initial synaptic dysfunction, and then by an increase in synaptic loss [137]. Ultimately, the combination of all the previous events may promote the cognitive impairments observed in behavioral tests [137].

## 7. Altered ATP Release from Astrocytes in AD

It is now well established that adenosine triphosphate (ATP) serves as an important neurotransmitter in the central and peripheral nervous system [138]. ATP mediates fast and slow synaptic potentials via ligand-gated cationic channels (P2X receptors) and G protein-coupled receptors (P2Y receptors). It is also now apparent that ATP is released from astrocytes as a gliotransmitter [139]. There has been a large body of evidence indicating multiple release mechanisms of ATP from astrocytes. Several studies have supported the notion that ATP is released from astrocytes in a calcium-dependent manner via exocytosis from synaptic-like vesicles [140,141,142,143,144]. Alternatively, several nonexocytotic release mechanisms also have been described for astrocytic ATP release. These include connexin hemichannels (connexin 43) [145], pannexin channels (Panx-1) [146], the calcium-dependent chloride channel bestrophin [141], and the calcium homeostasis modulator (CALHM) [147].

Enhancement of ATP release in hippocampal slices or astrocyte cultures is observed upon application of Aβ peptides [148]. In cultured rat astrocytes, exposure of astrocytes to Aβ1-42 increased the amplitude and velocity of evoked calcium waves mediated by enhanced ATP release [148]. Several studies have proposed that connexin 43 (Cx43) in a hemichannel function mediates the Aβ peptide induction of ATP release from astrocytes. Thus, Cx43 is upregulated in AD mouse models and AD human brains [149,150,151,152]. Moreover, amyloid exposure triggers the increased expression of Cx43 both in vitro and in vivo AD models [82,153,154]. Cx43 is also highly permeable to ATP [155], and knocking out the Cx43 gene in the APP/PPS1 mice reduces ATP release and decreases neuronal damage [140]. Finally, astrocytes of acute hippocampal slices containing Aβ plaques in the APP/PS1 mice show enhanced hemichannel activity and ATP release [82,88] (Figure 2).

ATP release from astrocytes in AD pathology may directly induce pathology. Conceivably, ATP activation of calcium-permeable pathways such as via P2X7 receptors or co-activation of NMDAR/P2X7 receptors could reduce the survival of neurons [82,135,136]. On the other hand, a protective role of ATP released from astrocytes in AD has also been suggested. Jung et al. found that exogenous ATP protects Aβ-mediated reduction of synaptic function in hippocampal neuronal cultures [156]. In addition, ATP prevented the Aβ-induced impairment of LTP in hippocampal slices [156].

## 8. Altered GABA Release from Astrocytes in AD

**Figure 3:**
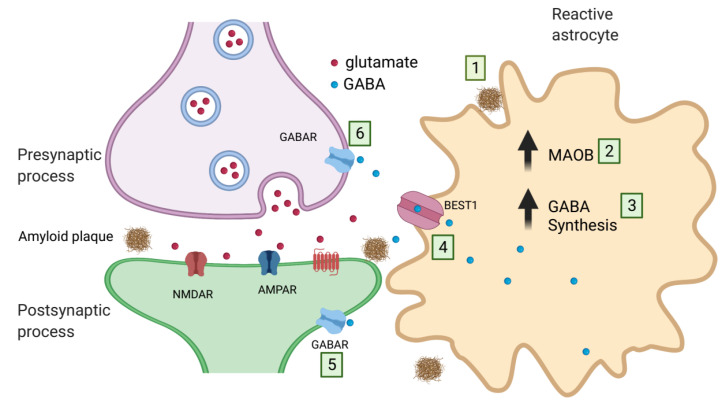
Schematic representation of GABA release from astrocytes in AD pathology. (1) Amyloid plaques evoke an enhanced activation of the monoamine oxidase MAOB (2) to induce the GABA synthesis using putrescine as a substrate (3). GABA is released from astrocytes via the anion channel BEST1 (4). GABA released as a gliotransmitter acts on postsynaptic (5) or presynaptic (6) GABA receptors (GABAR) to impact synaptic transmission. Arrows indicate increases.

GABA is the major inhibitory neurotransmitter in the adult mammalian brain, and recent studies have also shown astrocytic release as a gliotransmitter in AD [157]. In the healthy brain, cytosolic concentrations in astrocytes are kept at low levels. However, in human AD patients and mouse models of AD, the astrocytic GABA cytosolic levels are unusually high [158]. Atypically, astrocytes seem to synthesize GABA from putrescine as a substrate via monoamine oxidation instead of using glutamate as a substrate [159]. GABA released from astrocytes exert a tonic inhibitory influence onto cerebellar granule neurons and striatal medium spiny neurons via the activation of GABA_A_ receptors [159]. In the prefrontal cortex, on the other hand, astrocytic GABA interacts with GABAergic interneurons via GABA_B_ receptors [160]. Under normal conditions, hippocampal astrocytes contain very little GABA [161], but diseased astrocytes around amyloid plaques become reactive and aberrantly and abundantly produce and release GABA via the anion channel bestrophin 1 (BEST1) [161] (Figure 3). Behaviorally, GABA from reactive astrocytes inhibits the activity of dentate granule neurons, resulting in inhibition of spike probability and contributing to learning and memory impairments [161].

A large body of literature indicates that AD disproportionally affects women in both occurrence and severity [162,163]. Interestingly, in a transgenic mouse model of AD (Tg2576 mice), GABA levels in the hippocampus were substantially higher in females than males [164]. This extra GABA was produced via the monoamine oxidase-B from putrescine in reactive astrocytes [164]. Thus, the reactivity of astrocytes and their dysregulated release of GABA may contribute to the observed sex differences in AD.

## 9. Altered D-Serine Release from Astrocytes in AD

NMDA receptors (NMDARs) play a central role in synaptic plasticity and learning, and memory. A distinctive feature of these receptors is the requirement for a co-agonist binding to the GluN1 subunit with glutamate binding to its recognition site on the GluN2-3 subunits for the activation of the receptor [165]. While glycine was originally thought to be the co-agonist on NMDARs, several studies over the last few decades have demonstrated that D-serine is at least as important in modulating NMDARs [166,167].

Serine racemase (SR) synthesizes D-serine in the brain by converting L-serine to D-serine. D-serine is a putative gliotransmitter that has been linked to learning and memory by its actions on synaptic NMDARs [168]. The astrocytic release of D-serine has been linked to the induction of LTP in the hippocampus and hypothalamus [168]. Altered levels of D-serine have been associated with neurological disorders, including schizophrenia and epilepsy. Interestingly, D-serine levels in postmortem hippocampal and cortical samples of AD patients seem to be higher than in control patients [169].

Moreover, levels of both D-serine and serine racemase, the enzyme responsible, are also elevated in experimental models of AD [169,170]. Increased expression of serine racemase in reactive astrocytes in TgF344-AD rats was also associated with elevated extra-synaptic NMDAR signaling [170]. The increased levels of D-serine and NMDAR hyperfunction may contribute to memory impairments and excitotoxicity observed in AD. More recently, it has been reported that serum D-serine levels serve as a biomarker for the progression of AD [171].

## 10. Changes to Gliotransmission in Neurodegenerative Diseases Other Than AD

It is not unexpected that altered astrocyte–neuronal communication and gliotransmission are also found in neurodegenerative diseases other than AD. Indeed, impaired glutamate uptake by astrocytes is a well-documented dysfunction of astrocytes in Parkinson’s disease (PD), amyotrophic lateral sclerosis (ALS), and Huntington’s disease (HD) [172]. Diminished levels of GLT-1 expression are found in postmortem brain tissues from patients with ALS, PD, or HD [173,174]. As discussed above, dysregulation of expression and function of GLT-1 is linked to chronically high levels of extracellular glutamate causing excitotoxicity [174]. Therefore, pharmacological induced upregulation of GLT-1 in neurodegenerative diseases is a promising approach to treating chronic excitotoxicity. Likewise, the enhanced expression of astrocytic Cx43 is altered in animal models of ALS and PD [175]. As in AD, the high expression of Cx43 is hypothesized to cause excessive release of ATP or glutamate into the extracellular space [175], ultimately leading or contributing to neurodegeneration [176]. Thus, there are many shared features in astrocyte–neuronal communication changes in diverse types of neurodegenerative diseases that may be a consequence of the astrocytic responses to their reactive state. Finding the commonalities and differences in astrocyte–neuronal communication in various neurodegenerative diseases may allow the design of novel strategies to treat these diseases.

## 11. Concluding Remarks

**Figure 4:**
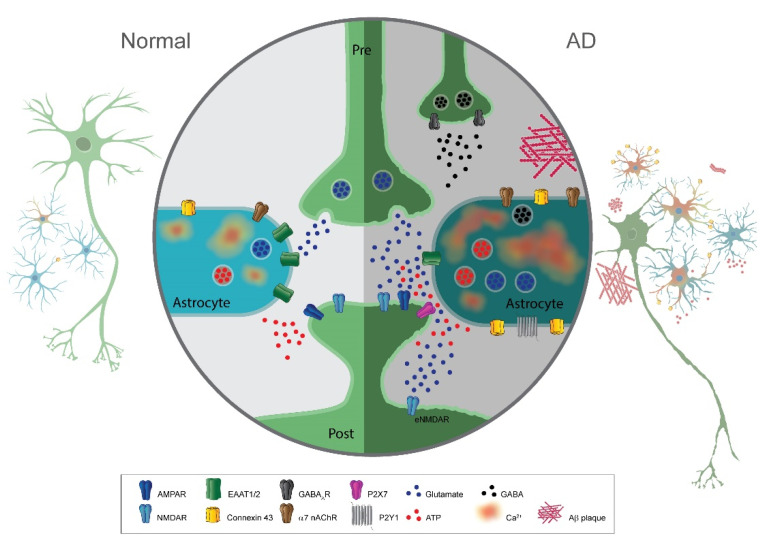
Summary of astrocyte–neuron communication alterations in Alzheimer´s disease. Representation of an astrocytic and neuronal network in the normal state (**left**) and during the pathogenesis of AD (**right**). The magnification in the middle depicts a tripartite synapse in the normal state (**left**) and AD (**right**). Neurons are colored in green and astrocytes are colored in blue. Spontaneous astrocytic calcium signals are augmented in AD. The increase in calcium activity has been suggested to be mediated through upregulation of the purinergic receptor P2Y1, α7 nAChR, and/or the metabotropic glutamate receptor mGluR5 in astrocytes. Elevations in astrocytic intracellular calcium concentration promote the up-regulation of Cx43, leading to enhanced release of gliotransmitters (glutamate, ATP, and GABA). Diseased astrocytes express lower amounts of the glutamate transporter EAAT2 (or GLT-1), contributing to the accumulation of glutamate in the synaptic cleft. The increase in glutamate and ATP will overstimulate ionotropic receptors with consequent neuronal death. Thus, the presence of Aβ and the malfunctioning of astrocytes in AD lead to impairments in the neuronal network, decreasing the density of synaptic puncta and the basal synaptic transmission, which in turn will produce impairments in learning and memory. AD: Alzheimer´s disease; α7 nAChR: α7 nicotinic acetylcholine receptors; Cx43: connexin 43; eNMDAR: extra-synaptic NMDA receptor.

The exquisite and complex electrochemical communication between astrocytes and neurons is now being deciphered by the ever more sophisticated imaging and molecular techniques. It has become increasingly evident that this bidirectional communication between glia and neurons has striking effects on the functioning of synapses. However, changes in this form of communication during disease have received little consideration. We would argue from the above-mentioned studies that considerable evidence has now been gathered from many laboratories that imply that this glia–neuron dialogue is altered in AD with consequences for neuronal network functions (Figure 4). This impaired communication at synapses may well turn out to be an important factor in the chain of events that lead to cognitive impairments observed in AD. From a translational point of view, astrocytes may then provide important targets for therapeutic strategies for the treatment of AD.

## Data Availability

Not applicable.
